# Measurement of the high-energy gamma-ray emission from the Moon with the Fermi Large Area Telescope

**DOI:** 10.1103/PhysRevD.93.082001

**Published:** 2016-04-08

**Authors:** M. Ackermann, M. Ajello, A. Albert, W. B. Atwood, L. Baldini, G. Barbiellini, D. Bastieri, R. Bellazzini, E. Bissaldi, R. D. Blandford, R. Bonino, E. Bottacini, J. Bregeon, P. Bruel, R. Buehler, G. A. Caliandro, R. A. Cameron, M. Caragiulo, P. A. Caraveo, E. Cavazzuti, C. Cecchi, A. Chekhtman, J. Chiang, G. Chiaro, S. Ciprini, R. Claus, J. Cohen-Tanugi, F. Costanza, A. Cuoco, S. Cutini, F. D’Ammando, A. de Angelis, F. de Palma, R. Desiante, S. W. Digel, L. Di Venere, P. S. Drell, C. Favuzzi, S. J. Fegan, W. B. Focke, A. Franckowiak, S. Funk, P. Fusco, F. Gargano, D. Gasparrini, N. Giglietto, F. Giordano, M. Giroletti, T. Glanzman, G. Godfrey, I. A. Grenier, J. E. Grove, S. Guiriec, A. K. Harding, J. W. Hewitt, D. Horan, X. Hou, G. Iafrate, G. Jóhannesson, T. Kamae, M. Kuss, S. Larsson, L. Latronico, J. Li, L. Li, F. Longo, F. Loparco, M. N. Lovellette, P. Lubrano, J. Magill, S. Maldera, A. Manfreda, M. Mayer, M. N. Mazziotta, P. F. Michelson, W. Mitthumsiri, T. Mizuno, M. E. Monzani, A. Morselli, S. Murgia, E. Nuss, N. Omodei, E. Orlando, J. F. Ormes, D. Paneque, J. S. Perkins, M. Pesce-Rollins, V. Petrosian, F. Piron, G. Pivato, S. Rainò, R. Rando, M. Razzano, A. Reimer, O. Reimer, T. Reposeur, C. Sgrò, E. J. Siskind, F. Spada, G. Spandre, P. Spinelli, H. Takahashi, J. B. Thayer, D. J. Thompson, L. Tibaldo, D. F. Torres, G. Tosti, E. Troja, G. Vianello, B. L. Winer, K. S. Wood, M. Yassine, Fermi LAT Collaboration, F. Cerutti, A. Ferrari, P. R. Sala

**Affiliations:** 1 Deutsches Elektronen Synchrotron DESY, D-15738 Zeuthen, Germany; 2 Department of Physics and Astronomy, Clemson University, Kinard Lab of Physics, Clemson, South Carolina 29634-0978, USA; 3 W. W. Hansen Experimental Physics Laboratory, Kavli Institute for Particle Astrophysics and Cosmology, Department of Physics and SLAC National Accelerator Laboratory, Stanford University, Stanford, California 94305, USA; 4 Santa Cruz Institute for Particle Physics, Department of Physics and Department of Astronomy and Astrophysics, University of California at Santa Cruz, Santa Cruz, California 95064, USA; 5 Università di Pisa and Istituto Nazionale di Fisica Nucleare, Sezione di Pisa I-56127 Pisa, Italy; 6 Istituto Nazionale di Fisica Nucleare, Sezione di Trieste, I-34127 Trieste, Italy; 7 Dipartimento di Fisica, Università di Trieste, I-34127 Trieste, Italy; 8 Istituto Nazionale di Fisica Nucleare, Sezione di Padova, I-35131 Padova, Italy; 9 Dipartimento di Fisica e Astronomia “G. Galilei”, Università di Padova, I-35131 Padova, Italy; 10 Istituto Nazionale di Fisica Nucleare, Sezione di Pisa, I-56127 Pisa, Italy; 11 Istituto Nazionale di Fisica Nucleare, Sezione di Bari, I-70126 Bari, Italy; 12 Istituto Nazionale di Fisica Nucleare, Sezione di Torino, I-10125 Torino, Italy; 13 Dipartimento di Fisica Generale “AmadeoAvogadro”, Università degli Studidi Torino, I-10125 Torino, Italy; 14 Laboratoire Univers et Particules de Montpellier, Université Montpellier, CNRS/IN2P3, F-34095 Montpellier, France; 15 Laboratoire Leprince-Ringuet, École polytechnique, CNRS/IN2P3, F-91128 Palaiseau Cedex, France; 16 Consorzio Interuniversitario per la Fisica Spaziale (CIFS), I-10133 Torino, Italy; 17 Dipartimento di Fisica “M. Merlin” dell’Università e del Politecnico di Bari, I-70126 Bari, Italy; 18 INAF-Istituto di Astrofisica Spaziale e Fisica Cosmica, I-20133 Milano, Italy; 19 Agenzia Spaziale Italiana (ASI) Science Data Center, I-00133 Roma, Italy; 20 Istituto Nazionale di Fisica Nucleare, Sezione di Perugia, I-06123 Perugia, Italy; 21 Dipartimento di Fisica, Università degli Studi di Perugia, I-06123 Perugia, Italy; 22 College of Science, George Mason University, Fairfax, Virginia 22030, USA and resident at Naval Research Laboratory, Washington, District of Columbia 20375, USA; 23 INAF Osservatorio Astronomico di Roma, I-00040 Monte Porzio Catone (Roma), Italy; 24 INAF Istituto di Radioastronomia, I-40129 Bologna, Italy; 25 Dipartimento di Astronomia, Università di Bologna, I-40127 Bologna, Italy; 26 Dipartimento di Fisica, Università di Udine and Istituto Nazionale di Fisica Nucleare, Sezione di Trieste, Gruppo Collegato di Udine, I-33100 Udine; 27 Università Telematica Pegaso, Piazza Trieste e Trento, 48, I-80132 Napoli, Italy; 28 Università di Udine, I-33100 Udine, Italy; 29 Erlangen Centre for Astroparticle Physics, D-91058 Erlangen, Germany; 30 Laboratoire AIM, CEA-IRFU/CNRS/Université Paris Diderot, Service d’Astrophysique, CEA Saclay, F-91191 Gif sur Yvette, France; 31 Space Science Division, Naval Research Laboratory, Washington, District of Columbia 20375-5352, USA; 32 NASA Goddard Space Flight Center, Greenbelt, Maryland 20771, USA; 33 NASA Postdoctoral Program Fellow, USA; 34 Department of Physics, University of North Florida, 1 UNF Drive, Jacksonville, Florida 32224, USA; 35 Yunnan Observatories, Chinese Academy of Sciences, Kunming 650216, China; 36 Key Laboratory for the Structure and Evolution of Celestial Objects, Chinese Academy of Sciences, Kunming 650216, China; 37 Osservatorio Astronomico di Trieste, Istituto Nazionale di Astrofisica, I-34143 Trieste, Italy; 38 Science Institute, University of Iceland, IS-107 Reykjavik, Iceland; 39 Department of Physics, Graduate School of Science, University of Tokyo, 7-3-1 Hongo, Bunkyo-ku, Tokyo 113-0033, Japan; 40 Department of Physics, KTH Royal Institute of Technology, AlbaNova, SE-106 91 Stockholm, Sweden; 41 The Oskar Klein Centre for Cosmoparticle Physics, AlbaNova, SE-106 91 Stockholm, Sweden; 42 Institute of Space Sciences (IEEC-CSIC), Campus UAB, E-08193 Barcelona, Spain; 43 Department of Physics and Department of Astronomy, University of Maryland, College Park, Maryland 20742, USA; 44 Department of Physics, Faculty of Science, Mahidol University, Bangkok 10400, Thailand; 45 Hiroshima Astrophysical Science Center, Hiroshima University, Higashi-Hiroshima, Hiroshima 739-8526, Japan; 46 Istituto Nazionale di Fisica Nucleare, Sezione di Roma “Tor Vergata”, I-00133 Roma, Italy; 47 Center for Cosmology, Physics and Astronomy Department, University of California, Irvine, California 92697-2575, USA; 48 Department of Physics and Astronomy, University of Denver, Denver, Colorado 80208, USA; 49 Max-Planck-Institut für Physik, D-80805 München, Germany; 50 Funded by contract FIRB-2012-RBFR12PM1F from the Italian Ministry of Education, University and Research (MIUR); 51 Institut für Astro- und Teilchenphysik and Institut für Theoretische Physik, Leopold-Franzens-Universität Innsbruck, A-6020 Innsbruck, Austria; 52 Centre d’Études Nucléaires de Bordeaux Gradignan, IN2P3/CNRS, Université Bordeaux 1, BP120, F-33175 Gradignan Cedex, France; 53 NYCB Real-Time Computing Inc., Lattingtown, New York 11560-1025, USA; 54 Department of Physical Sciences, Hiroshima University, Higashi-Hiroshima, Hiroshima 739-8526, Japan; 55 Max-Planck-Institut für Kernphysik, D-69029 Heidelberg, Germany; 56 Institució Catalana de Recerca i Estudis Avançats (ICREA), E-08010 Barcelona, Spain; 57 Department of Physics, Center for Cosmology and Astro-Particle Physics, The Ohio State University, Columbus, Ohio 43210, USA; 58 European Organization for Nuclear Research (CERN), CH-1211 Geneva, Switzerland; 59 Istituto Nazionale di Fisica Nucleare, Sezione di Milano, I-20133 Milano, Italy

## Abstract

We have measured the gamma-ray emission spectrum of the Moon using the data collected by the Large Area Telescope onboard the Fermi satellite during its first seven years of operation, in the energy range from 30 MeV up to a few GeV. We have also studied the time evolution of the flux, finding a correlation with the solar activity. We have developed a full Monte Carlo simulation describing the interactions of cosmic rays with the lunar surface. The results of the present analysis can be explained in the framework of this model, where the production of gamma rays is due to the interactions of cosmic-ray proton and helium nuclei with the surface of the Moon. Finally, we have used our simulation to derive the cosmic-ray proton and helium spectra near Earth from the Moon gamma-ray data.

## I. INTRODUCTION

The Moon, as well as other bodies in the solar system, can be passive sources of high-energy gamma rays, resulting from inelastic collisions of energetic cosmic-ray (CR) particles with their material [1]. A measurement of the lunar gamma-ray flux therefore represents a useful tool to investigate the properties of CRs outside Earth’s magnetic field. Such a study does require accurate modeling of the interaction processes of high-energy CRs with the lunar surface.

The emission of high-energy gamma rays from the Moon was first observed by the EGRET experiment [2], which operated from 1991 to 2000 on the Compton Gamma Ray Observatory. More precise results were recently published by the Fermi LAT Collaboration using the data collected by the Large Area Telescope (LAT) during its first two years of operation [3], which provided a measurement of the gamma-ray flux above 100 MeV.

In the present work, we have evaluated the gamma-ray flux from the Moon using the data collected by the Fermi LAT in its first seven years of operation, from August 2008 to June 2015. Not only is the current data set much larger, but the data were processed with the newest Pass 8 reconstruction and event-level analysis [4], allowing the useful energy range to be extended well below 100 MeV. We have studied the time evolution of the gamma-ray flux from the Moon, finding the expected correlation with the solar activity.

Gamma rays from the Moon are mainly emitted with sub-GeV energies, and their flux depends on the fluxes of CRs impinging on the Moon and on their inelastic interactions with the lunar regolith. The chemical composition of the lunar surface also plays a crucial role in determining the gamma-ray yield. As will be discussed in Sec. VI, the energy spectrum of lunar gamma rays is sensitive to the spectra of CR primaries in the range up to a few tens of GeV/n, which are strongly affected by the solar activity.

Therefore, the main ingredients of any model aiming to provide an interpretation of the gamma-ray emission from the Moon are (a) the interactions of CRs with matter, (b) the lunar surface composition, and (c) the CR energy spectra. The models describing inelastic interactions of CRs with matter are well validated against the data from accelerator experiments and are quite reliable in the energy range of interest. The predicted gamma-ray spectra will therefore depend on the input CR spectra and on the lunar surface composition.

Simultaneous measurements of the lunar gamma-ray spectrum and of the spectra of charged CRs near Earth can provide the possibility to test the chemical composition of the lunar surface. In fact, the CR energy spectra provided as input to the models are usually evaluated from the data collected in a different epoch and accounting for solar modulation. The simultaneity allows eliminating uncertainties on the CR spectra due to solar modulation. The AMS-02 instrument is currently taking data simultaneously with the Fermi LAT, and recently its measurements of the CR proton and helium energy spectra near Earth have been published [5,6]. This fact therefore offers the unprecedented possibility to set severe constraints on the lunar gamma-ray emission models.

In this work, we have implemented a full Monte Carlo simulation of the CR interactions with the Moon surface based on the FLUKA [7–9] code. In our simulation, we assumed a lunar surface chemical composition derived from the samples of lunar rock taken by the astronauts of the Apollo missions [10]. We show that the simulation reproduces accurately the Moon gamma-ray data taken by the LAT in the same epoch as the AMS-02 proton and helium data. Finally, starting from a model of the local interstellar spectra (LIS) of CR protons and helium nuclei, we have fitted the Moon gamma-ray data using the gamma-ray yields predicted by our simulation to derive the CR proton and helium spectra at 1 AU from the Sun and to evaluate solar modulation potential.

## II. LUNAR GAMMA-RAY EMISSION SPECTRUM

As mentioned in Sec. I, gamma rays emitted from the Moon are produced after inelastic interactions of charged CRs with the lunar surface. Hereafter, we will make the assumption that the CR flux on the lunar surface is spatially isotropic.

Indicating with *I*_*i*_(*T*) the intensity of CRs of the *i*th species (in units of particles MeV^−1^ cm^−2^ sr^−1^ s^−1^) as a function of kinetic energy *T*, the rate Γ_*i*_(*T*)of CRs of the *i*th species (in units of particles MeV^−1^ s^−1^) impinging on the lunar surface will be given by

(1) $$\Gamma_{i}(T)=4\pi R_{☾}^{2}I_{i}(T)\int \text{cos}\theta_{M}d\Omega_{M}=4\pi^{2}R_{☾}^{2}I_{i}(T),$$

where *R*_☾_ = 1737.1 km is the radius of the Moon. In the previous equation, we set *d*Ω_*M*_ = *d* cos *θ*_*M*_*dϕ*_*M*_, where (*θ*_*M*_, *φ*_*M*_) are the zenith and azimuth angles of CR particles with respect to the lunar surface (0 < cos *θ*_*M*_ < 1 and 0 < *ϕ*_*M*_ < 2*π*).

The differential gamma-ray luminosity of the Moon *L*_*γ*_(*E*_*γ*_) (in units of photons MeV^−1^ s^−1^) is given by

(2) $$L_{\gamma }\left(E_{\gamma }\right)=\sum_{i}\int Y_{i}\left(E_{\gamma }|T\right)\Gamma_{i}(T)dT\;=4\pi^{2}R_{☾}^{2}\sum_{i}\int Y_{i}\left(E_{\gamma }|T\right)I_{i}(T)dT,$$

where *Y*_*i*_(*E*_*γ*_|*T*) is the differential gamma-ray yield (in units of photons particle^−1^ MeV^−1^), i.e. the number of photons per unit energy produced by a primary particle of the *i*th species. The yields *Y*_*i*_(*E*_*γ*_|*T*) depend on the mechanisms of interactions of primary CRs with the lunar surface (regolith) and on its composition.

The differential intensity of gamma rays (in units of photons MeV^−1^ cm^−2^ sr^−1^ s^−1^) emitted from the Moon can be evaluated starting from the differential luminosity and is given by

(3) $$I_{\gamma }\left(E_{\gamma }\right)=\frac{L_{\gamma }\left(E_{\gamma }\right)}{4\pi^{2}R_{☾}^{2}}=\sum_{i}\int Y_{i}\left(E_{\gamma }|T\right)I_{i}(T)dT.$$

The gamma-ray flux observed by a detector at Earth (in units of photons MeV^−1^ cm^−2^ s^−1^) can also be evaluated from the differential luminosity and is given by

(4) $$\phi_{\gamma }\left(E_{\gamma }\right)=\frac{L_{\gamma }\left(E_{\gamma }\right)}{4\pi d^{2}}=\frac{\pi R_{☾}^{2}}{d^{2}}I_{\gamma }\left(E_{\gamma }\right)\;=\frac{\pi R_{☾}^{2}}{d^{2}}\sum_{i}\int Y_{i}\left(E_{\gamma }|T\right)I_{i}(T)dT,$$

where *d* is the distance between the center of the Moon and the detector. In the case of the Fermi LAT, due to the orbital motions of the Moon and of the Fermi satellite around the Earth, *d* ranges from about 3.4 × 10^5^ km to 4.1 × 10^5^ km (i.e. from about 54*R*_⊕_ to 64*R*_⊕_, where *R*_⊕_ = 6378 km is the mean equatorial Earth radius).

In particular, Eq. (4) shows that a 10% change of the distance *d* corresponds to a 20% change of the flux. This effect cannot be eliminated from our data analysis because, due to the limited photon statistics, in order to properly reconstruct the fluxes, we need to analyze data samples collected in periods of at least a few months, which are longer than the time scales corresponding to the orbital periods of the Moon (~28 days) and of the LAT (~1.5 h).

## III. DATA SELECTION

The LAT is a pair conversion gamma-ray telescope, sensitive in the energy range from 20 MeV to more than 300 GeV. Here, a brief description of the instrument is given, while full details can be found in Ref. [11].

The instrument is a 4 × 4 array of 16 identical towers, designed to convert incident gamma rays into *e*^+^*e*^−^ pairs and to determine their arrival directions and energies. Each tower is composed of a tracker module and a calorimeter module. The tracker consists of 18 *x* − *y* planes of silicon strip detectors interleaved with tungsten converter foils, for a total on-axis thickness of 1.5 radiation lengths. The calorimeter consists of 96 CsI (Tl) crystals, hodoscopically arranged in eight layers. The towers are surrounded by a segmented anticoincidence detector consisting of plastic scintillators, which is used for rejecting the charged cosmic-ray background.

The analysis presented in this paper has been performed using the newest Pass 8 data [4], specifically P8_SOURCE photon events starting from a minimum energy of 30 MeV.

A crucial point in the Moon gamma-ray data analysis is the treatment of the background, which originates variously from the diffuse gamma-ray emission, from the gamma-ray sources that the Moon drifts past along its path in the sky, and from the tiny residual fraction of charged CRs that are misclassified as photons. As the Moon is a moving source, the use of a background template might lead to inaccurate results. Hence, for our analysis, we chose to evaluate the background directly from the data, by using properly selected signal and background regions.

The signal region is defined as a cone centered on the Moon position, with an energy dependent angular radius given by

(5) $$\theta =\sqrt{{\left[\theta_{0}{\left(E/E_{0}\right)}^{-\delta }\right]}^{2}+\theta_{\text{min}}^{2}},$$

where *E* is the photon energy, *E*_0_ = 100 MeV, *θ*_min_ = 1°, *θ*_0_ = 5°, and *δ* = 0.8. The energy dependence of the angular radius follows the behavior of the 68% containment radius of the LAT point-spread function (PSF) [12]. This choice maximizes the signal-to-noise ratio. The value of *θ*_min_ in Eq. (5) has been chosen to account for the finite dimension of the Moon, which is seen from the Earth as an extended source of 0.25° angular radius. The position of the Moon is obtained from its ephemeris using software interfaced to the JPL libraries [13] and correcting for Fermi orbital parallax.

The background region is a cone of the same angular radius as the signal region, centered on a time-offset position of the Moon. Since the Moon orbits around the Earth with a period of ~28 days, we chose a time offset of 14 days (i.e. at a given time, the center of the background region is in the position that the Moon will take 14 days later). We performed our analysis by splitting the data set in smaller subsamples, each of 1 month duration. This means that in a month of 30 days, the center of the background region will take 16 days to reach the position occupied by the Moon at the end of that month. When this happens, the center of the background region will be brought back to the position taken by the Moon at the beginning of the month, and starting from this time, it will move along the path described by the Moon during the first 14 days of the month. In this way, the background region will span the same portion of sky as the signal region, and since the orbital period of the Moon is close to 1 month, the angular separation between the centers of the signal and background regions will always be close to 180°.

For the analysis of the signal (and background) region, we selected the time intervals when the LAT was operating in its standard science operation configuration and was outside the South Atlantic Anomaly. To avoid contamination from the bright limb of the Earth, we discarded the data taken during the times when the angular separation between a cone of angular radius *θ*_max_ = 15° centered on the Moon^1^ direction and the zenith direction exceeded 100°. We also discarded data taken during the times when the Moon was observed with off-axis angles *θ* larger than 66.4° (i.e. cos *θ* < 0.4). To mitigate the systematic uncertainties due to the bright diffuse gamma-ray emission from the Galactic plane, in our analysis we selected only the periods where the Moon was at a Galactic latitude |*b*_☾_| > 20°. We also required a minimum angular distance of 20° between the Moon and the Sun and between the Moon and any bright^2^ celestial source in the second Fermi LAT source catalog (2FGL) [14]. Since the center of the background region spans the same portion of sky as the Moon and the good time intervals for the two regions are chosen in the same way, the exposures of the signal and of the background regions are nearly identical.

## IV. DATA ANALYSIS AND RESULTS

**FIG. 1.**
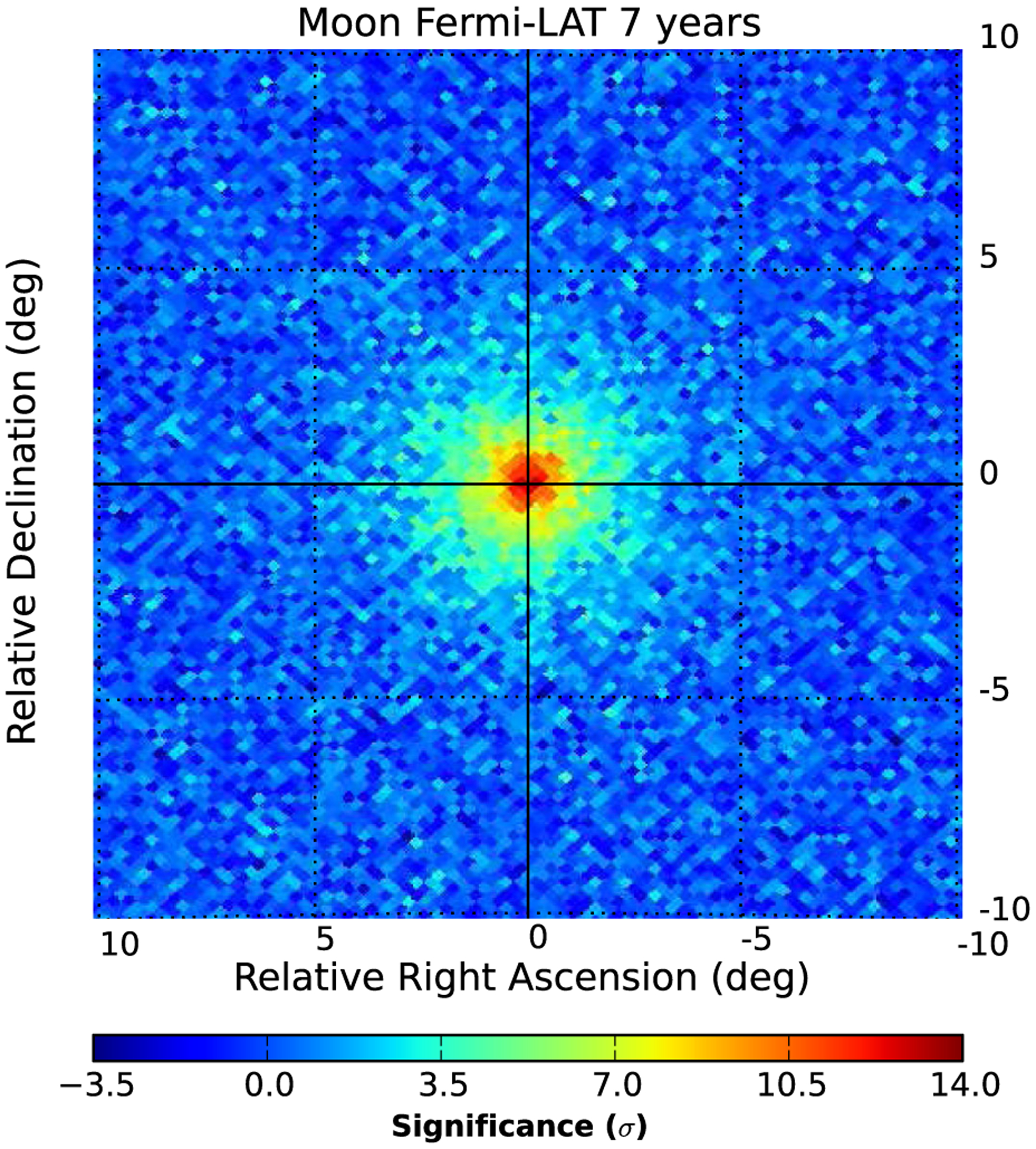
Significance map of the Moon as a function of right ascension and declination relative to the instantaneous Moon position for photons in the energy range from 30 MeV to 10 GeV. The map is built using a HEALPix [16] pixelization of the sky with *N*_side_ = 256 (each pixel corresponds to a solid angle ≈1.6 × 10^−5^ sr). The significance is evaluated following the prescriptions of Ref. [15].

Figure 1 shows the significance map of the gamma-ray signal from the Moon. The map has been built selecting photons with energies from 30 MeV to 10 GeV. The significance of each pixel has been evaluated according to the prescriptions of Ref. [15], starting from the counts in the signal and in the background regions and taking into account the live time ratio between the two regions. As expected, the significance map exhibits a clear peak in its center, corresponding to the gamma-ray emission from the Moon. The angular size of the peak is broader than that of the lunar disk (0.25°) due to the finite PSF of the LAT and is comparable with the value of the PSF at 200 MeV (2.9°), where the peak of the signal count spectrum is found.

**FIG. 2.**
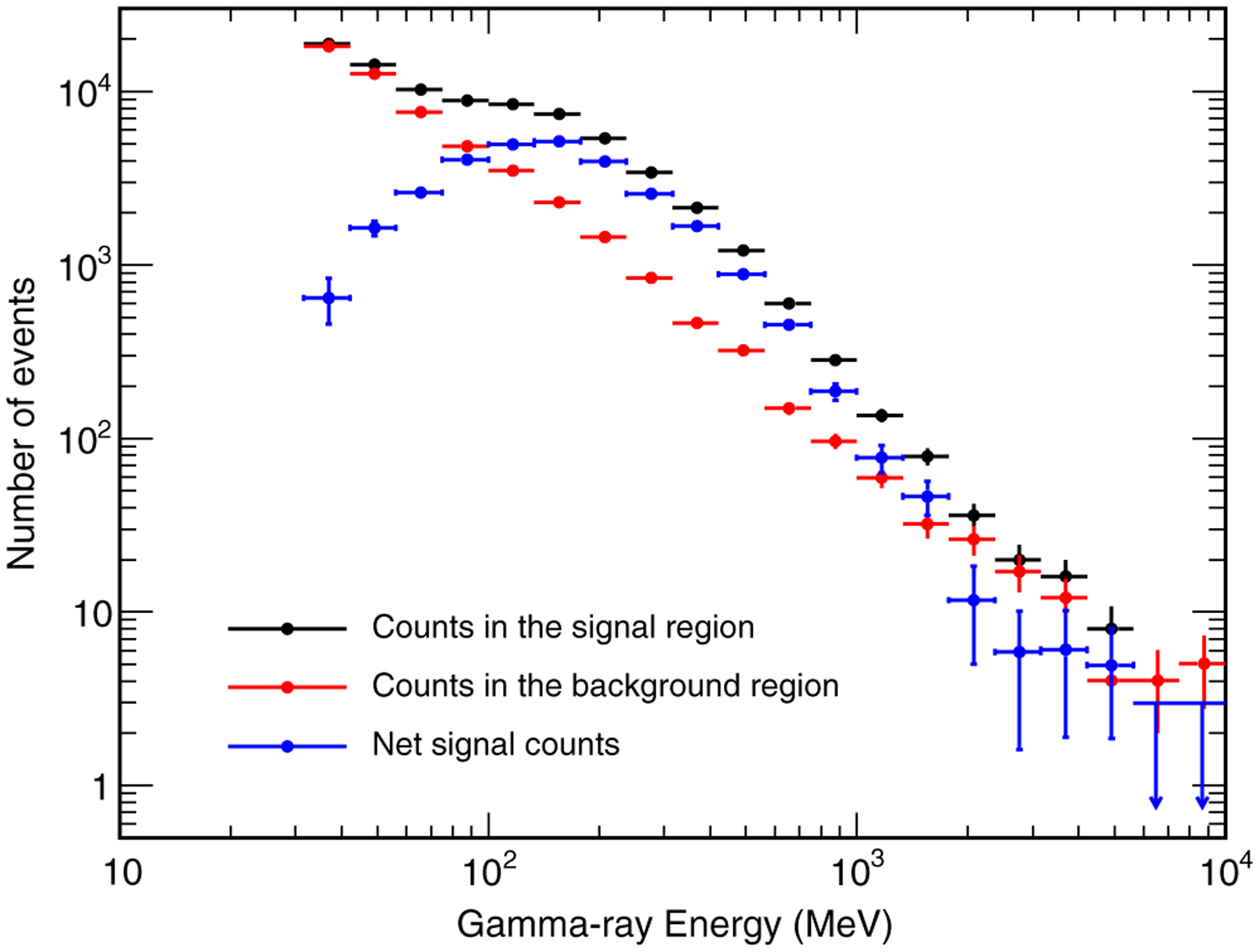
Count distributions as a function of gamma-ray energy for the signal (black circles) and background (red circles) regions. Blue symbols represent the net signal count spectrum, evaluated by the method described in Ref. [17]. Circles and associated error bars represent the average values and the rms values of the corresponding PDFs. Arrows represent upper limits at 95% confidence level.

Figure 2 shows the observed photon count spectra in the signal and background regions and the net signal count spectrum. The latter was calculated by applying in each energy bin the Bayesian procedure illustrated in Ref. [17], taking into account the live times of the signal and background regions and assuming uniform priors for the net signal counts in each energy bin. In particular, for each energy bin we evaluated the posterior probability density function (PDF) for the signal counts. The central values of the net signal count spectrum shown in Fig. 2 represent the average values of the corresponding PDFs, while the error bars represent the corresponding rmss. In the energy bins where the significance of the net signal counts is smaller than 2*σ*, upper limits at 95% confidence level are shown.

To reconstruct the energy spectrum of gamma rays from the Moon starting from the observed count spectra and taking energy dispersion into account, we have implemented an analysis method based on the software toolkit BAT [18]. The BAT package allows evaluating the full posterior probability PDFs for the parameters of a model. It is based on Bayes’ theorem and is realized with the use of a Markov chain Monte Carlo (MCMC) analysis. In the present work, we used BAT to extract, starting from the observed count distributions in the signal and background regions, the posterior PDFs for both the signal and background gamma-ray fluxes.

Indicating with *μ*_*s*_(*E*_*i*_) and *μ*_*b*_(*E*_*i*_), the expected counts in the *i*th energy bin, respectively in the signal and in the background region, it is possible to write the following equations:

(6) $$\mu_{s}\left(E_{i}\right)=\sum_{j}P_{s}\left(E_{i}|E_{j}\right)\left[\phi_{s}\left(E_{j}\right)+\phi_{b}\left(E_{j}\right)\right]At_{s}\Delta E_{j}$$

(7) $$\mu_{b}\left(E_{i}\right)=\sum_{j}P_{b}\left(E_{i}|E_{j}\right)\phi_{b}\left(E_{j}\right)At_{b}\Delta E_{j}.$$

In the previous equations, *ϕ*_*s*_(*E*_*j*_) and *ϕ*_*b*_(*E*_*j*_) are the true signal and background fluxes in the jth energy bin [*ϕ*_*s*_(*E*) corresponds to *ϕ*_*γ*_(*E*) in Eq. (4)], that are treated as unknown parameters; *P*_*s*_(*E*_*i*_|*E*_*j*_) and *P*_*b*_(*E*_*i*_|*E*_*j*_) are the smearing matrices in the signal and background regions respectively, i.e. the probabilities that a photon of energy *E*_*j*_ is observed with energy *E*_*i*_, and are evaluated from a full Monte Carlo simulation of the instrument, taking into account the pointing histories of the two regions; *A* = 6 m^2^ is the cross sectional area of the spherical surface used for the generation of the events in the Monte Carlo simulation; *t*_*s*_ and *t*_*b*_ are the live times of the signal and background regions respectively.

If *n*_*s*_(*E*_*i*_) and *n*_*b*_(*E*_*i*_) are the actual values of the counts in the *i*th energy bin of the signal and of the background regions, it is possible to define the likelihood function as a product of Poisson PDFs,

(8) $$L\left({\vec{\phi }}_{s},{\vec{\phi }}_{b};{\vec{n}}_{s},{\vec{n}}_{b}\right)=\prod_{i}e^{-\mu_{s}\left(E_{i}\right)}\frac{\mu_{s}{\left(E_{i}\right)}^{n_{s}\left(E_{i}\right)}}{n_{s}\left(E_{i}\right)!}\times \prod_{i}e^{-\mu_{b}\left(E_{i}\right)}\frac{\mu_{b}{\left(E_{i}\right)}^{n_{b}\left(E_{i}\right)}}{n_{b}\left(E_{i}\right)!},$$

where we used the vector notation to denote sets of independent quantities defined in the various energy bins [i.e. ${\vec{\phi }}_{s}=\left(\phi_{s}\left(E_{1}\right),\phi_{s}\left(E_{2}\right),\ldots ,\right)$ etc.].

As the point for the MCMC, we assumed uniform prior PDFs for the unknown parameters *ϕ*_*s*_(*E*_*j*_) and *ϕ*_*b*_(*E*_*j*_). The posterior PDFs for *ϕ*_*s*_(*E*_*j*_) and *ϕ*_*b*_(*E*_*j*_) are evaluated by BAT using the likelihood function in Eq. (8).

**FIG. 3.**
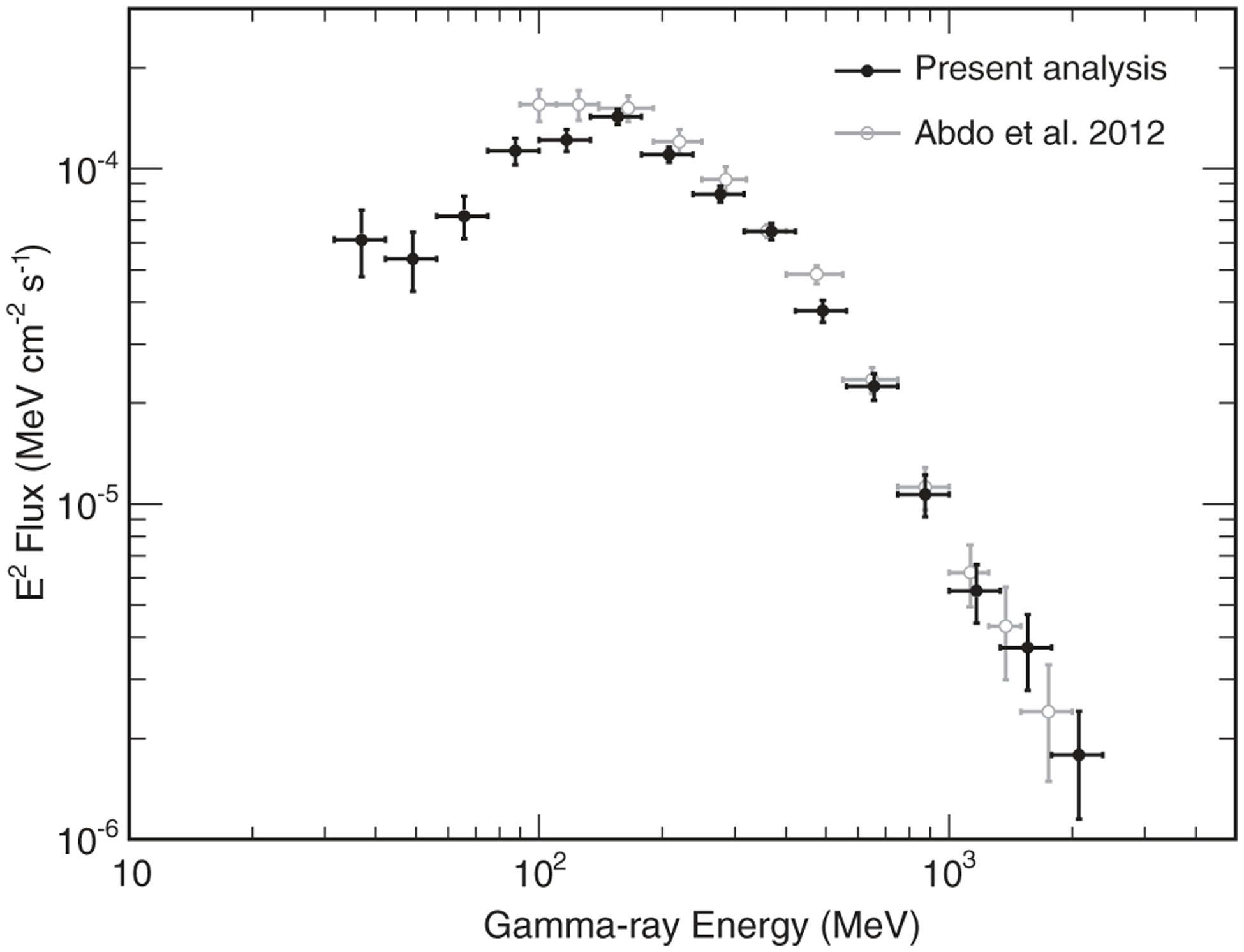
Gamma-ray energy spectrum of the Moon. The flux values *ϕ*_*γ*_(*E*) in each bin are multiplied by *E*^2^ = *E*_1_*E*_2_, where *E*_1_ and *E*_2_ are the lower and upper energy edges of each bin. The results from the present analysis (black points) are compared with those published in Ref. [3]. Only statistical error bars are shown. The central values of each bin represent the mean flux values, while the error bars represent the rmss of the corresponding PDFs.

Figure 3 shows the reconstructed gamma-ray spectrum of the Moon. The present results are compared with those published in Ref. [3], obtained from the analysis of the first 2 years of data taken by the Fermi LAT. The points shown in the plot correspond to the mean values of the PDFs on the signal fluxes in each bin, while the error bars indicate the rms values. The spectral energy distribution *E*^2^*ϕ*_*γ*_(*E*) is peaked at about 150 MeV and then drops with increasing energy as a power law with a spectral index of about −2.

The present results are consistent with those of Ref. [3] at energies above 150 MeV. The minor discrepancies in the range below 150 MeV can be ascribed to the solar modulation effect on CRs, which affects the energy spectrum of gamma rays emitted from the Moon (see also the discussion in Sec. V). The 2 years interval analyzed in Ref. [3] corresponded to the minimum of solar activity at the beginning of Solar Cycle 24. On the other hand, the data set used in this analysis spans a period of 7 years, covering more than half of Cycle 24. As a sanity check, we applied the analysis technique illustrated in this paper to the data taken by the LAT in the first 2 years, and the results were consistent with those of Ref. [3] in the whole energy range.

In Fig. 3, only statistical error bars on the fluxes are shown. The systematic uncertainties, not shown in Fig. 3, are primarily due to the uncertainties on the effective area of the instrument, which propagate to the gamma-ray fluxes. The uncertainties on the effective area were evaluated by the Fermi LAT Collaboration [19]: they drop from 10% to 3% in the energy range from 30 to 100 MeVand are ~3% at energies above 100 MeV. Systematic uncertainties are smaller than statistical ones in the whole energy range; in fact, the latter are of ~25% at 30 MeV, drop to ~5% at 150 MeV, and increase again to ~25% at 1.5 GeV.

To search for possible issues in the analysis, in addition to the approach discussed above and based on BAT, we implemented two more analysis techniques, and we compared the results.

In the first approach, we used the software toolkit MINUIT [20] to evaluate the set of parameters ${\vec{\phi }}_{s}$ and ${\vec{\phi }}_{b}$ that maximize the likelihood function in Eq. (8). We find that the results from the MINUIT analysis are consistent with those shown in Fig. 3 within the statistical errors in the whole energy range.

In the second approach, we used an improved version of the Bayesian unfolding technique originally developed by the Fermi LAT Collaboration for the spectral analysis of gamma-ray sources [21–24], in which we implemented the prescriptions of Ref. [25]. The starting point for the unfolding procedure is the set of posterior PDFs for the signal counts in each energy bin, which are used to build a set of random realizations of the signal count spectra. These count spectra are then unfolded, and the corresponding gamma-ray flux spectra are obtained. Finally, starting from these spectra, the PDFs on the fluxes in the various energy bins are evaluated. The results from the unfolding analysis are also consistent within the statistical errors with those shown in Fig. 3.

## V. TIME EVOLUTION STUDIES

To study the time evolution of the gamma-ray emission from the Moon, we performed the same analysis described in Sec. IV on subsets of data corresponding to 6 month intervals aligned with the beginning of the solar years (i.e. January to June and July to December except for the first one, starting in August 2008).

**FIG. 4.**
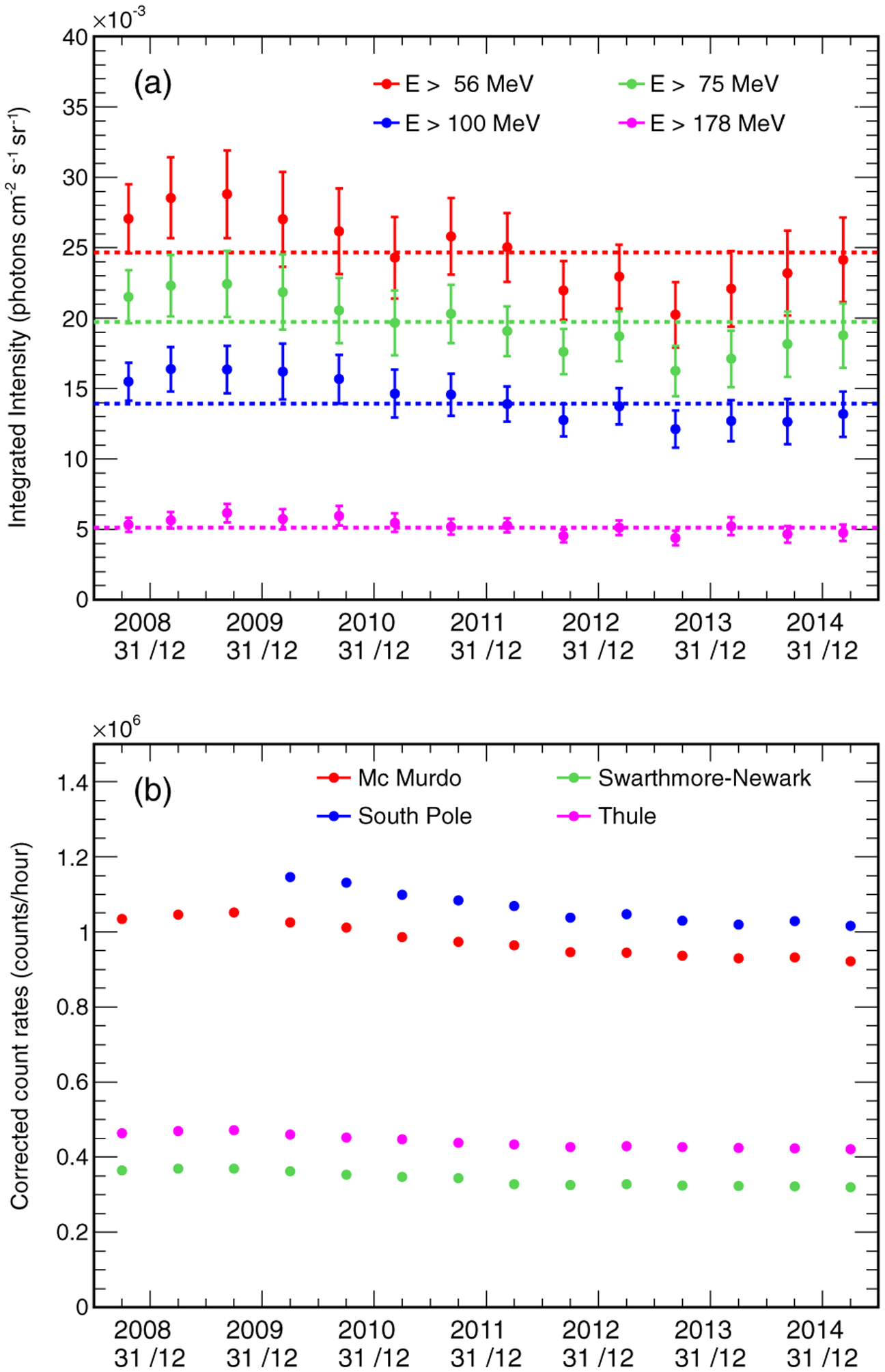
(a) Time evolution of the gamma-ray intensity from the Moon. The red, green, blue, and purple symbols represent the intensites above 56, 75, 100, and 178 MeV respectively. The dashed lines indicate the average values calculated over the whole data-taking period. (b) Time evolution of the corrected count rates registered by the neutron monitors of McMurdo (red), Newark (green), South Pole (blue), and Thule (purple). The data of the neutron monitors correspond to the good time intervals selected for the Moon data analysis. Each point of the plot corresponds to an average value taken over a 6 month period.

Figure 4(a) shows the time evolution of the gamma-ray intensities from the Moon above 56, 75, 100, and 178 MeV. The integral intensity is evaluated by integrating the differential intensity; the latter is calculated from the flux taking the LAT-Moon distance into account. The error bars shown in the figure have been calculated taking into account the statistical uncertainties on the fluxes and the variations of the distance between the LAT and the Moon during each data-taking period (see the discussion in Sec. II). The intensities in the different periods are compared with the averages, which are calculated considering the whole data-taking period.

Figure 4(b) shows the time evolution of the count rates registered by some neutron monitors of the Bartol Research Institute [26] installed in various locations in the northern (Thule and Newark) and southern (McMurdo and South Pole) hemispheres. The count rates are corrected for differences in atmospheric pressure. We selected only the neutron monitor data taken during the good time intervals selected for the analysis of the Moon (see the discussion in Sec. III). The data from the South Pole neutron monitor do not cover the whole LAT data-taking period because it was closed from November 2005 until February 2010.

**FIG. 5.**
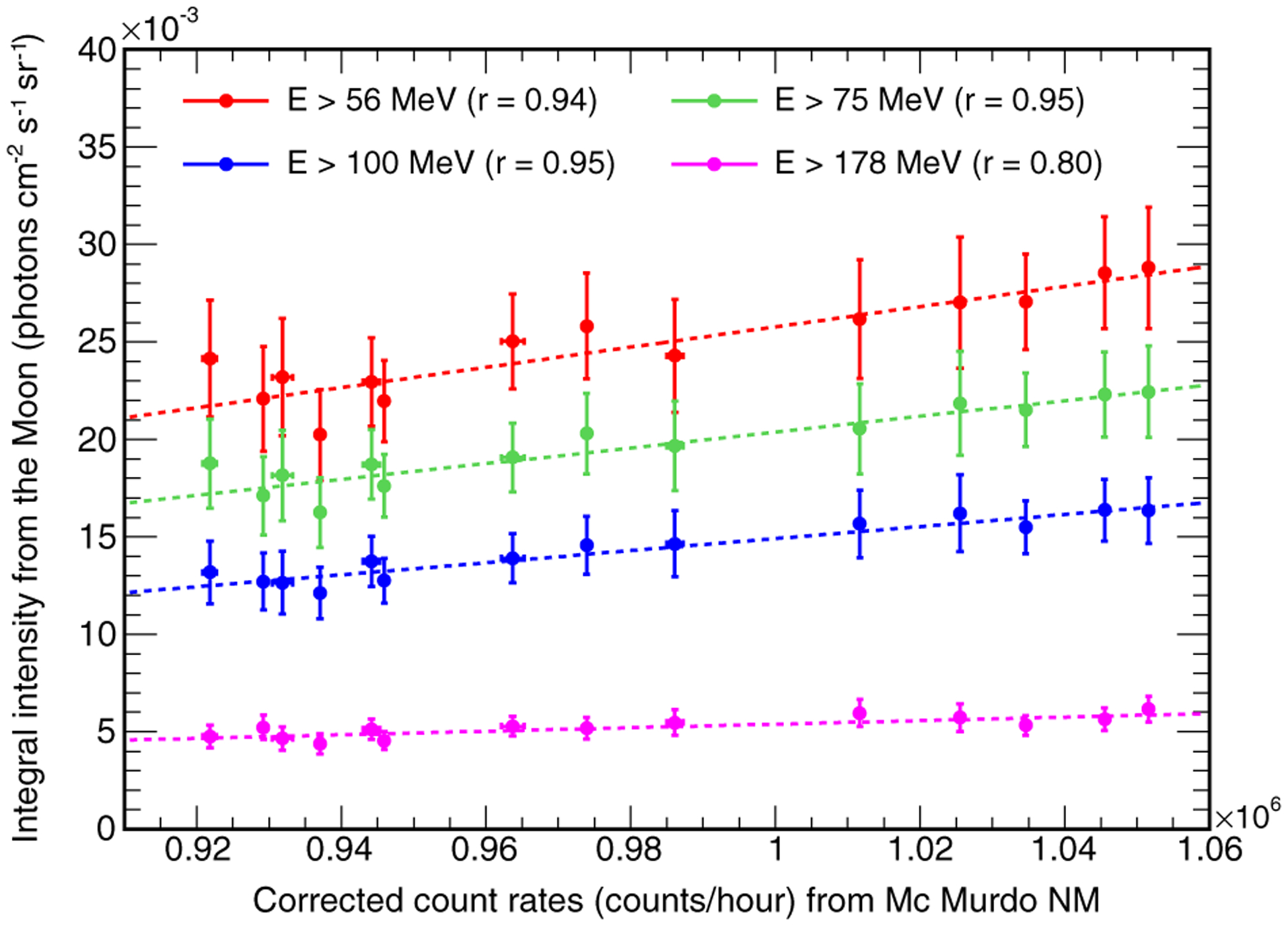
Comparison between the gamma-ray integral intensities from the Moon above 56 (red), 75 (green), 100 (blue), and 178 MeV (purple) and the count rate registered by the McMurdo neutron monitor. The dashed lines represent the linear regression curves of each series. The values reported in brackets are the correlation coefficients.

A comparison of the time evolution plots in Fig. 4 suggests that the gamma-ray emission of the Moon is correlated to the counts of the various neutron monitors. In Fig. 5, we plot the gamma-ray intensities from the Moon above 56, 75, 100, and 178 MeV against the count rates registered by the McMurdo neutron monitor. The data indicate that the lunar gamma-ray emission is indeed correlated with the neutron monitor count rate. In particular, the correlation is stronger when the gamma-ray energy threshold is lower and becomes weaker as the threshold increases. Similar results are obtained when comparing the lunar gamma-ray fluxes with the count rates registered by other neutron monitors. This result is expected, since gamma rays are produced in the interactions of primary CRs with the surface of the Moon, and therefore their flux must be affected by solar modulation. The correlation is more evident at low energies, because the solar modulation affects mainly the fluxes of low-energy CRs. In particular, in the case of CR protons, the effect is relevant at kinetic energies *T* ≲ 1–10 GeV. Since the typical energies of gamma rays produced in CR proton interactions are roughly one order of magnitude less than those of primary protons, the solar modulation effect is relevant for photons with energies *E*_*γ*_ ≲ 0.1 − 1 GeV.

## VI. MONTE CARLO SIMULATION OF CR INTERACTIONS WITH THE MOON

We have implemented a full Monte Carlo simulation of the interactions of CRs with the surface of the Moon based on the FLUKA [7–9] simulation code. This simulation has been used to evaluate the yields of gamma rays produced in these interactions.

FLUKA is a general-purpose Monte Carlo code for the simulation of hadronic and electromagnetic interactions. It is used in many applications and is continuously checked using the available data from low-energy nuclear physics, high-energy accelerator experiments, and measurements of particle fluxes in the atmosphere. Hadronic interactions are treated in FLUKA following a theory-driven approach. The general phenomenology is obtained from a microscopic description of the interactions between the fundamental constituents (quarks and nucleons), appropriate for the different energy ranges. Below an energy of a few GeV, the hadron-nucleon interactions model is based on resonance production and decay, while for higher energies the dual parton model is used. The extension from hadron-nucleon to hadron-nucleus interactions is done in the framework of the preequilibrium approach to nuclear thermalization (PEANUT) model [27,28], including the Gribov-Glauber multicollision mechanism followed by the preequilibrium stage and eventually equilibrium processes (evaporation, fission, Fermi breakup, and gamma deexcitation). In case of nucleus-nucleus interactions (in the present work involving alpha projectiles), DPMJET-III [29] and a modified version [30] of RQMD [31–33] are used as external event generators, depending on the projectile energy. More details about the FLUKA package can be found in the manual [8,9], and a description of hadronic interaction models used in FLUKA can be found in Ref. [34].

We have calculated the gamma-ray yields from the Moon assuming two different composition models for the lunar surface. To test these models, we have used the Moon gamma-ray data taken in the same period as the AMS-02 proton and helium data [5,6]. We have folded the CR proton and helium spectra measured by AMS-02 with the gamma-ray yields predicted by the simulation, and we have compared the resulting predicted fluxes with the data. Having found good agreement between the model and the data for one of the surface composition models, we have assumed a model for the LIS of CR protons and helium nuclei, and starting from the Moon gamma-ray data, we have evaluated the solar modulation potential in the framework of the force field approximation.

### A. Evaluation of the gamma-ray yield from the Moon

As mentioned in Sec. I, in any calculation of the lunar gamma-ray emission, a Moon surface model must be assumed, which includes a description of its geometry and its chemical composition. Regarding the geometry, in our simulation we made the simplest assumption that the Moon is a perfect sphere of radius *R*_☾_ = 1737.1 km, thus neglecting the roughness of the lunar surface (the top of the highest mountain and the bottom of the deepest crater are within ±10 km from the surface) and its eccentricity (the difference between the equatorial radius and the polar radius is <3 km).

About the chemical composition, we note that the available data are from actual samples of lunar rock taken by the astronauts in the different landing sites of the Apollo missions and from the low-energy gamma-ray, alpha, and neutron spectroscopy data [10]. Over the years, many models of the lunar surface have been proposed. In particular, for the present work, we adopted the lunar surface models proposed by Moskalenko and Porter in 2007 [35] (which was also used in Ref. [3]) and by Turkevich in 1973 [36] (hereafter, these models will be indicated in the text as “MP” and “TUR”). The features of the MP and TUR models are summarized in Table I. The main differences between the two models can be found in the weight fractions of the different oxides and in the density of the lunar surface. The differences result in a lighter composition (lower average atomic and mass numbers) of the TUR model with respect to the MP model.

For both models, we have evaluated the gamma-ray yield from the Moon by simulating protons and ^4^He nuclei with different kinetic energies impinging isotropically on the lunar surface. The kinetic energies are taken on a grid of 81 equally spaced values in logarithmic scale from 100 MeV/n to 10 TeV/n. The gamma-ray yield from the *i*th species of CR primaries (here *i* = *p*, ^4^He) *Y*_*i*_(*E*_*γ*_*|T*) is calculated as

(9) $$Y_{i}\left(E_{\gamma }|T\right)=\frac{N_{\gamma ,i}\left(E_{\gamma }|T\right)}{N_{i}(T)\Delta E_{\gamma }},$$

where *N*_*i*_(*T*) is the number of primaries of the *i*th species generated with kinetic energy *T* and *N*_*γ*,*i*_(*E*_*γ*_|*T*) is the number of photons with energies between *E*_*γ*_ and *E*_*γ*_ + Δ*E*_*γ*_ produced by the primaries of the type *i* with energy *T* and escaping from the surface of the Moon.

**FIG. 6.**
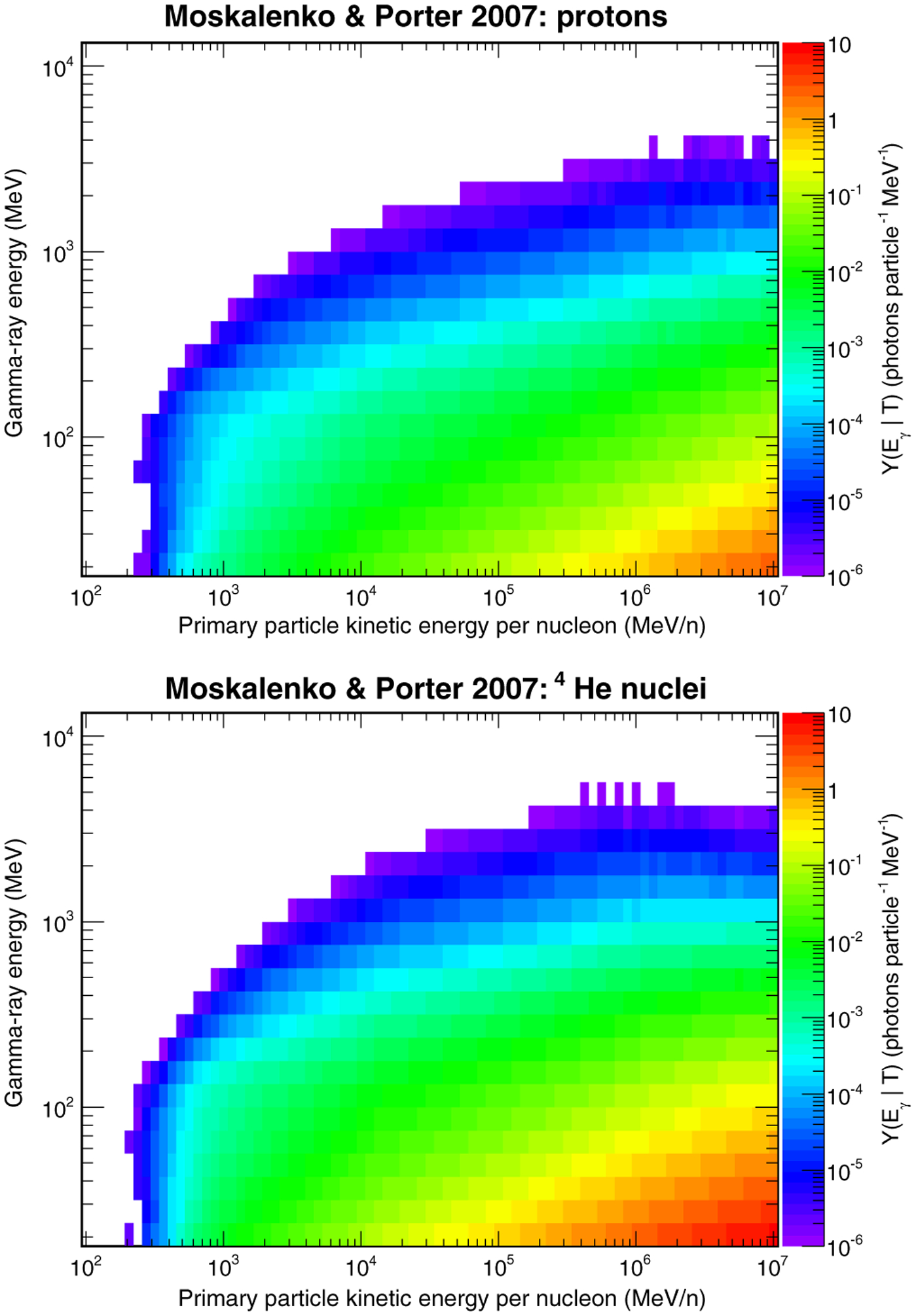
Yields of gamma rays produced by the interactions of protons (top) and ^4^He nuclei (bottom) on the Moon. The yields have been evaluated assuming the MP composition model.

Figure 6 shows the gamma-ray yields from the interactions of primary protons and ^4^He nuclei with the Moon calculated with the FLUKA simulation as a function of the kinetic energy per nucleon of the primary and of the gamma-ray energy assuming the MP composition model. From these plots, it is evident that, for both proton and ^4^He primaries, the gamma-ray yield is negligible for *T=n* ≲ 200 MeV=n. This is because most gamma rays originate from the decays of neutral pions, and the process of *π*^0^ production in *p*-nucleus and ^4^He-nucleus interactions requires a threshold kinetic energy for the incident particle.

**FIG. 7.**
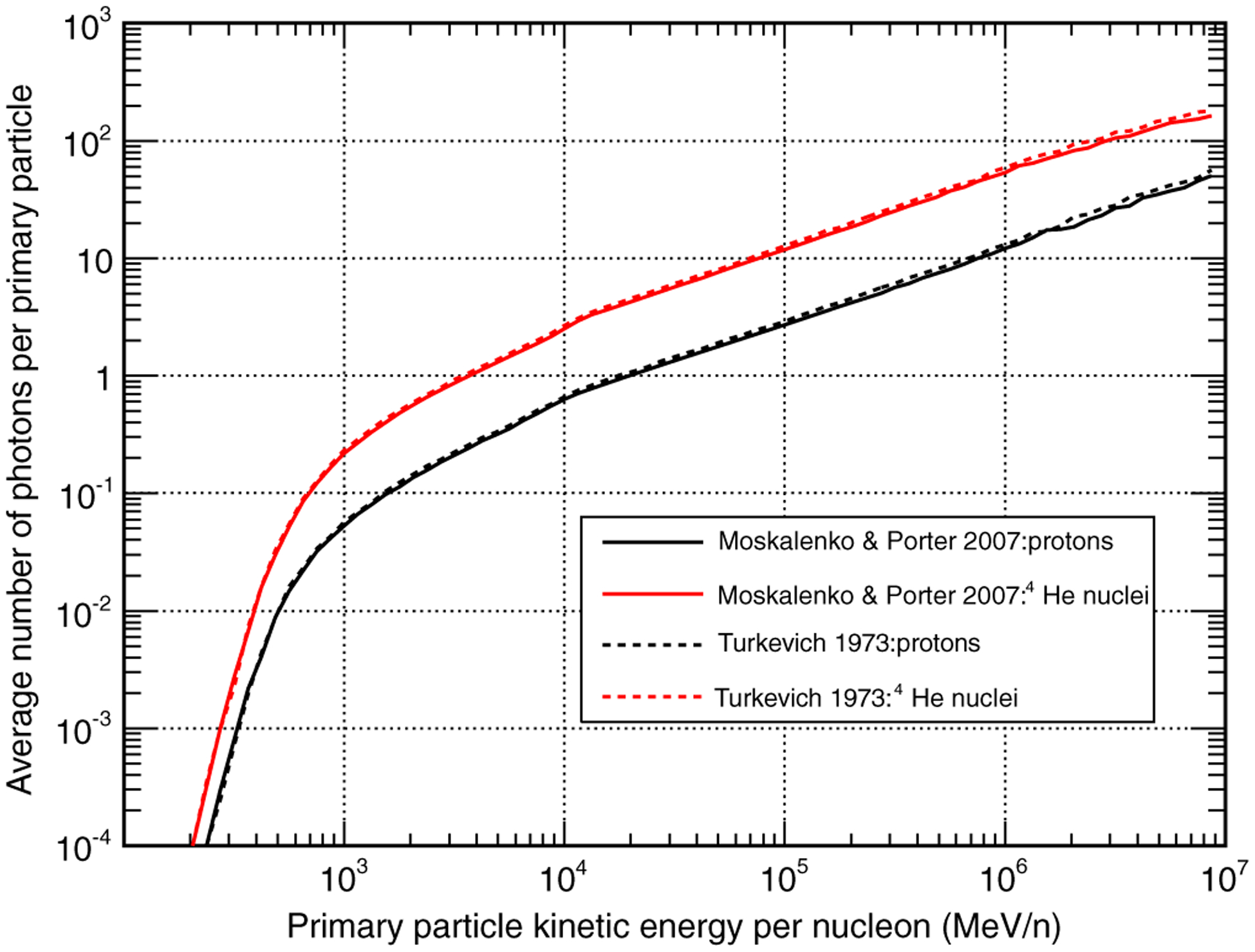
Average number of gamma rays per primary particle (in units of photons/particle) produced by primary protons (black) and ^4^He nuclei (red) as a function of the primary particle kinetic energy per nucleon. The calculations have been performed for both the MP (continuous lines) and the TUR (dashed lines) composition models.

Figure 7 shows the average number of photons per primary particle as a function of the projectile kinetic energy per nucleon produced by protons and ^4^He nuclei, calculated assuming the MP and TUR composition models. As can be seen in the figure, a ^4^He nucleus produces on average about four times more gamma rays than a proton with the same kinetic energy per nucleon. A simple interpretation of this fact can be given in terms of the superposition model, according to which a ^4^He nucleus is equivalent to four nucleons.

Another interesting result is that the gamma-ray yields predicted by the MP and TUR models are quite similar. Indeed, a deeper inspection of the results shows that the yields calculated with the TUR model are about 20% higher than those calculated with the MP model. The differences could be due either to the different compositions or to the different densities. To test a possible dependence of the gamma-ray yield on the density, we performed some simulations with the TUR and with the MP models keeping the composition unchanged and changing the density. The results showed that the gamma-ray yield is almost independent of the density. We can therefore conclude that the gamma-ray yield is mainly determined by the chemical composition of the lunar surface. In particular, the results suggest that higher values of 〈*Z*〉 and 〈*A*〉 correspond to lower gamma-ray yields

In both these models, the lunar surface composition is assumed to be independent of depth. Recently, another lunar surface model, based on the neutron and gamma-ray data from the Lunar Prospector mission, was proposed by Ota *et al.* [37], in which the regolith composition and density are assumed to change with depth. In particular, in the Ota model, the lunar surface is described as a stack of four different layers, each with different thicknesses, compositions, and densities (the details of this model are given in Table I of Ref. [37]). The gamma-ray yields calculated with the Ota model, not shown in the figure, are intermediate between those calculated with the MP and TUR models. This result was expected, since the values of 〈*Z*〉 and are 〈*A*〉 for all the layers composing the lunar surface intermediate between those of the MP and TUR models.

### B. Evaluation of the lunar gamma-ray spectrum

To evaluate the lunar gamma-ray intensity spectrum, we should fold the spectra of the various species of CRs impinging on the lunar surface with the gamma-ray yields calculated from the Monte Carlo simulation according to Eq. (3). In our calculation, we will consider only the contributions from protons and 4He nuclei, neglecting those from heavier nuclei. This approximation turns out to be reasonable when taking into account the relative abundances of the various CR species. Following the considerations in the previous section, we can roughly assume that the gamma-ray yields from different nuclei are proportional to the number of their constituent nucleons. Hence, assuming that the relative abundance of CR ^4^He nuclei with respect to protons is ~10%, the contribution of ^4^He nuclei to the lunar gamma-ray emission is expected to be ~40% of the proton contribution and therefore cannot be neglected. On the other hand, if we assume a relative abundance of carbon nuclei with respect to protons of ~0.1%, we expect their contribution to the lunar gamma-ray emission to be ~1% of the proton contribution. Since other CR components are even less abundant than carbon, we can conclude that the errors from neglecting heavier CR species in the calculation of the lunar gamma-ray spectrum will be of the order of a few percent.

We also emphasize here that in the calculation of the lunar gamma-ray spectrum the isotopic composition of primary CRs should be taken into account. However, in the following, we will assume that all CRs with *Z* = 1 are protons and all CRs with *Z* = 2 are ^4^He nuclei. Recent measurements [38] performed by the PAMELA experiment show that the ^2^H/^1^H ratio decreases from 3.5% to 1.8% in the energy range from 0.1 up to 1 GeV/n, while the ^3^He/^4^He ratio increases from about 8% up to 18% in the same energy range. Since deuterons and 3He are secondaries produced in the interactions of primary CRs with the interstellar medium, it is reasonable to think that their abundances do not increase significantly at higher energies. Therefore, assuming these values for the isotopic ratios, we expect that the error on the lunar gamma-ray spectrum calculated neglecting the isotopic composition of primary CRs will be of percent order.

The contribution to the differential gamma-ray intensity of the Moon from the *i*th species of CR projectiles (protons and ^4^He nuclei) may be calculated as

(10) $$\frac{dI_{\gamma ,i}\left(E_{\gamma }|T\right)}{dT}=Y_{i}\left(E_{\gamma }|T\right)I_{i}(T).$$

The corresponding photon energy flux can be then evaluated as

(11) $$E_{\gamma }^{2}\frac{d\Phi_{\gamma ,i}\left(E_{\gamma },T\right)}{dT}=E_{\gamma }^{2}\frac{\pi R_{☾}}{d^{2}}\frac{dI_{\gamma ,i}\left(E_{\gamma }|T\right)}{dT}.$$

**FIG. 8.**
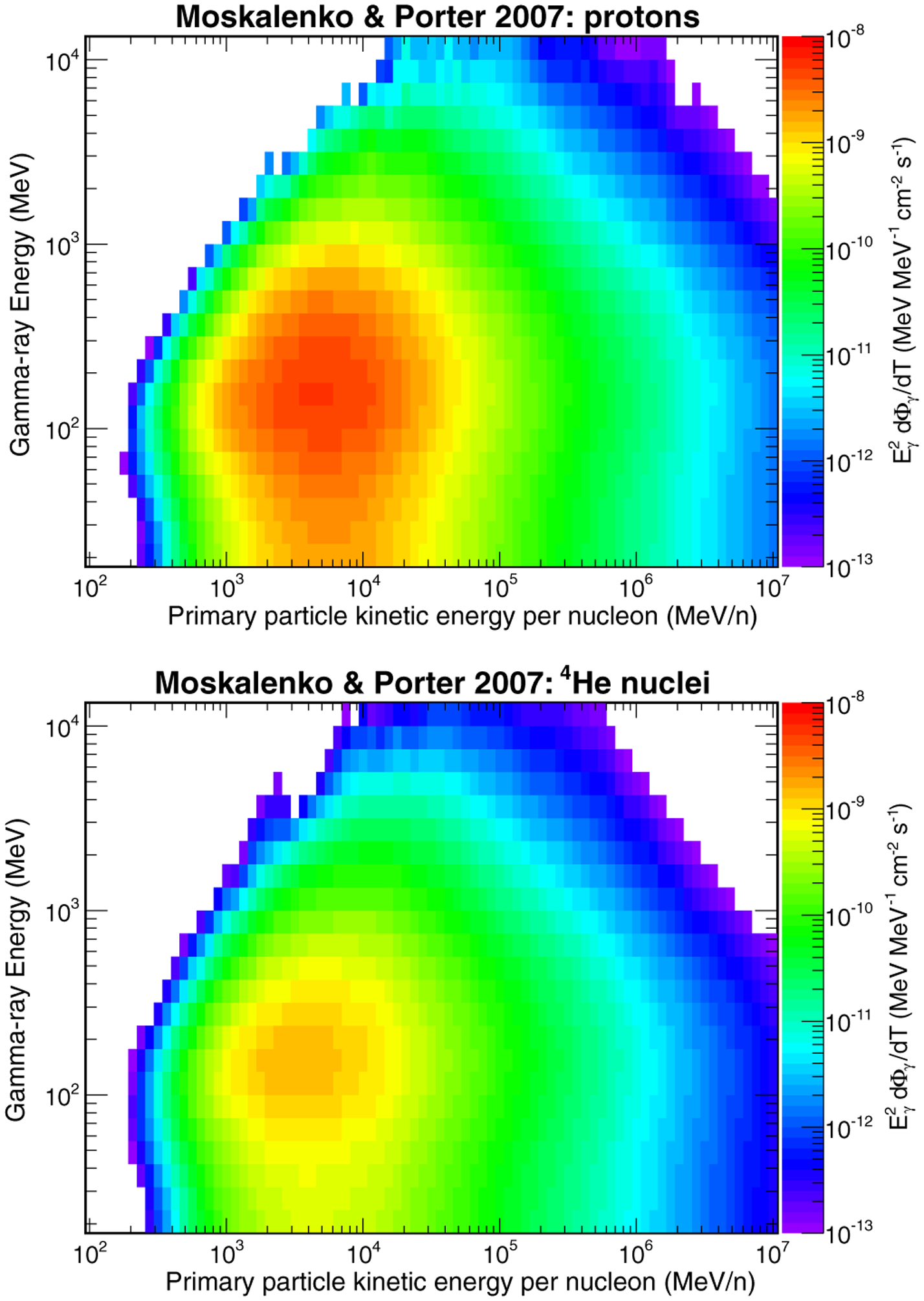
Differential photon energy flux from the Moon produced by the interactions of protons (top) and 4He nuclei (bottom) with the Moon surface. The photon intensities have been evaluated by folding the gamma-ray yields with the CR proton and helium intensity spectra measured by AMS-02 [5,6]. The calculation has been performed with the Moon surface composition model in Ref. [35].

Figure 8 shows, for the MP lunar composition model, the differential gamma-ray energy fluxes originated by proton and ^4^He primaries. The calculations have been performed by folding the proton and helium intensity spectra *I*_*p*_(*T*) and *I*_*He*_(*T*) measured by AMS-02 [5,6] with the gamma-ray yields calculated with our simulation.^3^ The calculations show that, although the gamma-ray yield increases with increasing primary energy, the contribution of high-energy primaries (*T* > 100 GeV in the case of protons) to the lunar gamma-ray emission is negligible, due to the shape of the primary intensity spectra (at high energies, *I*_*p*_(*T*) ~ *T*^−2.7^, and a similar behavior is observed for helium primaries). On the other hand, the main contribution to the lunar gamma-ray emission comes from primaries with energies in the range from about 1 GeV/n up to a few tens of GeV/n.

### C. Comparison of the Moon gamma-ray data with the predictions from direct observations of the CR proton spectrum

As mentioned in Sec. I, the data set used for this analysis was taken in a time interval spanning the whole data-taking period of AMS-02 [5,6]. This provides, for the first time, the possibility to test our Monte Carlo simulation against the direct measurements of the CR proton and helium spectra performed by AMS-02. Our data set is also partially overlapping with the data-taking period of PAMELA. However, at present, a test of the simulation against the PAMELA data is not possible. In fact, although the PAMELA Collaboration measured the CR proton spectra on monthly basis until the end of 2009 [39], they did not perform similar measurements of the helium spectra.

To test our simulation against the AMS-02 data, we selected a data sample taken in the period from May 2011 to November 2013. However, it is worthwhile to point out here that the time intervals selected for our analysis of the gamma-ray emission from the Moon most likely do not match those used for the AMS-02 data analysis in Ref. [5]. In particular, when applying the event selection described in Sec. III, we disregarded those time intervals corresponding to transient events, such as solar flares, that might be included in the AMS data analysis.

We then folded the CR proton and helium reference spectra with the gamma-ray yields obtained from our simulation with the MP and TUR models. When evaluating the gamma-ray flux, we assumed the LAT-Moon distance equal to its average value during the data-taking period from May 2011 to November 2013. In our calculations, we did not take into account the uncertainties on the proton and helium spectra measured by AMS-02, which are of about 2% on average [5,6].

**FIG. 9.**
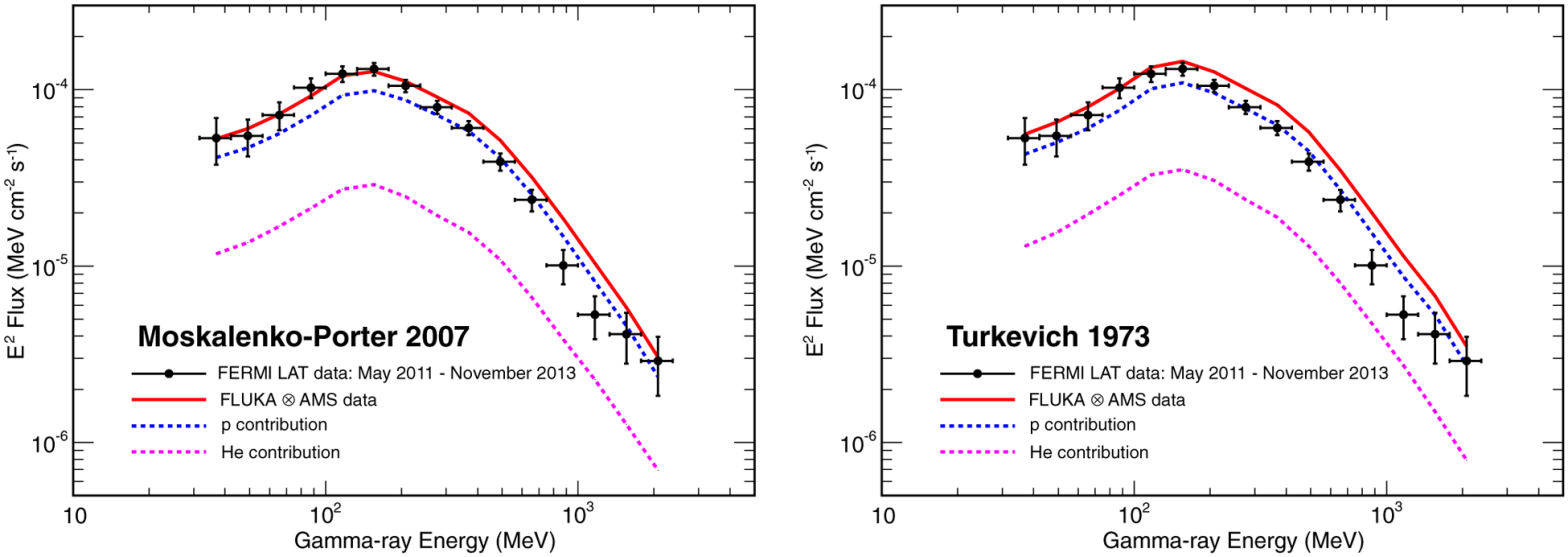
Gamma-ray flux from the Moon as a function of energy in the period May 2011–November 2013. The results from the LAT data analysis (black points) are compared with the expected fluxes obtained after folding the CR proton and helium spectra measured by AMS-02 in 2011–13 with the gamma-ray yields evaluated in Sec. VI A with our simulation. The calculations were perfomed using the lunar surface composition models in Refs. [35] (left) and [36] (right). The continuous red lines indicate the total flux, while the dashed blue and purple lines represent the contributions to the lunar gamma-ray spectrum from protons and helium nuclei respectively.

Figure 9 compares the measured gamma-ray fluxes with the calculations from the Monte Carlo simulation for the two composition models. As shown in the figure, the gamma-ray spectrum obtained from the MP composition model reproduces quite well the data in the whole energy range, with small discrepancies in the region around 1 GeV, where the observed flux is smaller than predicted. On the other hand, the spectrum obtained from the TUR composition model seems to slightly overestimate the data in the energy range above 200 MeV. According to the discussion in Sec. VI A, this result can be attributed to the relatively lighter regolith (lower 〈*Z*〉 and the greater 〈*A*〉) in the TUR model and consequently gamma-ray yield.

We remark here that, when comparing the data with the model predictions, one should also take into account all the uncertainties, such as those originating from the fluctuations on the LAT-Moon distance (see Sec. II), those on the instrument effective area (see Sec. IV), those on the AMS proton and helium spectra (see the discussion above), and those on the hadronic interactions models. All these uncertainties are likely of 10% or less.

On the basis of this result, in the following discussion we will adopt the MP composition model for the lunar surface. The small discrepancies between the simulation and the data could be ascribed to inaccuracies in our model of CR interactions with the Moon. In our model, we assume that CR protons of all energies are impinging isotropically on the whole Moon surface. However, low-energy CRs could be affected by the Earth’s magnetic field in their journey to the Moon, in contrast with the hypothesis of an isotropic CR flux. In addition, in our model we describe the lunar surface as a uniform sphere, without accounting for the real morphology of the Moon. On the other hand, the implementation of a more detailed model would require a huge effort that is beyond the scope of the present work.

### D. Evaluation of the low-energy CR proton and ^4^He spectra and of the solar modulation potential

The data shown in Sec. V indicate that the lunar gamma-ray spectrum is sensitive to the solar modulation effect. This is because, as discussed in Sec. VI A, the main contribution to the gamma-ray spectrum of the Moon is that of CRs in the energy range up to ~10 GeV/n. In the present section, we will illustrate an application of our Monte Carlo simulation to the study of the solar modulation potential.

**FIG. 10.**
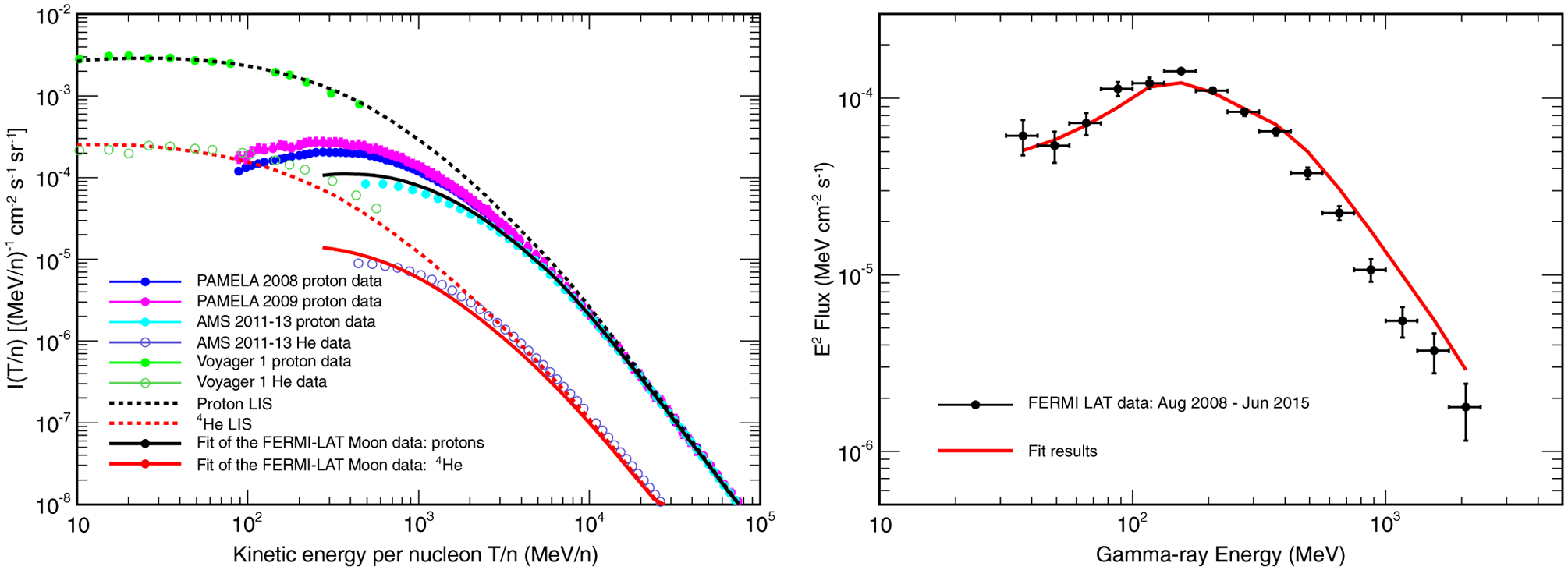
Left panel: CR proton and helium spectra obtained from the best fit of the Fermi LAT Moon gamma-ray data. The fit was performed using the MP lunar surface model. The results of the fit (continuous black and red lines) are compared with the proton measurements taken by PAMELA [39] in 2008 (blue points) and 2009 (purple points) and with the AMS-02 proton [5] (cyan points) and helium data [6] (violet points). The plot shows also the proton and helium LIS (dashed black and red lines) and the Voyager 1 proton (light green points) and helium (dark green) data [44]. Right panel: Gamma-ray flux from the Moon as a function of energy. The results from our analysis are compared with those of the fit. The continuous red line represents the average gamma-ray spectrum obtained from the fit, assuming that the Moon-LAT distance is equal to its average value during the whole data-taking period.

We start from a model for the CR proton and ^4^He LIS [40,41], evaluated using a customized version of the CR propagation code DRAGON [42,43], in which we included a set of cross sections for the production of secondary particles in CR interactions calculated with FLUKA. Both the proton and ^4^He LIS of Refs. [40,41] were derived in a general framework and, together with the LIS of other primary CR components, when propagated to the solar system, allow reproducing a wide set of observables. In particular, these observables include the measurements of CR protons performed by PAMELA [39] in 2008 and 2009, the measurements of CR protons and He nuclei performed by AMS-02 [5,6] from 2011 to 2013, and those performed by Voyager 1 [44] during its journey outside the Solar System. The proton and ^4^He LIS are shown in the left panel of Fig. 10, where they are also compared with the data from direct measurements. We emphasize here that at high energies the ^4^He LIS lies below the points measured by AMS-02 because, as mentioned in Sec. VI B, the AMS-02 data include both the ^4^He and ^3^He component.

In the following analysis, the intensity spectra *I*_*i*_(*T*) of the various CR species (protons and ^4^He nuclei) in the Solar System are evaluated starting from the LIS intensity spectra $I_{i}^{\text{LIS}}\left(T\right)$ using the force field approximation [45],

(12) $$I_{i}(T)=I_{i}^{\text{LIS}}\left(T+e\Phi Z_{i}/A_{i}\right)\times \frac{T\left(T+2m_{i}\right)}{\left(T+e\Phi Z_{i}/A_{i}\right)\left(T+e\Phi Z_{i}/A_{i}+2m_{i}\right)},$$

where *m*_*i*_, *Z*_*i*_, and *A*_*i*_ are the mass, the charge, and the number of nucleons of the *i*th primary component; *e* is the absolute value of the electron charge; and Φ is the solar modulation potential, which in the following discussion will be treated as a free parameter.

We used the proton and ^4^He LIS and the gamma-ray yields calculated with the MP composition model for the lunar surface to perform a fit of the data. The fit procedure is based on BAT and is similar to the one described in Sec. IV for the reconstruction of the gamma-ray fluxes from the Moon. In this case, the gamma-ray signal fluxes in the various energy bins are correlated and are calculated from the cosmic-ray proton and helium intensities *I*_*p*_(*T*) and *I*_*He*_(*T*) using Eqs. (2) and (4). Here, the parameters to be fitted are the background photon fluxes ${\vec{\phi }}_{b}$ and the solar modulation potential Φ. In our calculations, we assumed that the LAT-Moon distance *d*, that appears in Eq. (4), is constant and equal to its average value during the whole data-taking period.

The fitting procedure, applied to the whole 7 years data sample, yields a solar modulation potential of 537 ± 12 MV. The left panel of Fig. 10 shows the fitted CR proton and helium intensity spectra, compared with the results of the direct measurements performed by PAMELA and by AMS-02. As shown in the figure, the CR proton spectum inferred from this analysis is consistent with the results from direct measurements and lies between the PAMELA and the AMS-02 data. The helium spectrum lies below the AMS-02 data because, as discussed above, it includes only the ^4^He component.

The gamma-ray spectrum obtained from the fit is shown in the right panel of Fig. 10, where it is compared with the results from the data analysis discussed in Sec. IV. The fitted spectrum accurately reproduces the data in the energy range up to 400 MeV, while at higher energies it tends to overestimate the measured fluxes.

**FIG. 11.**
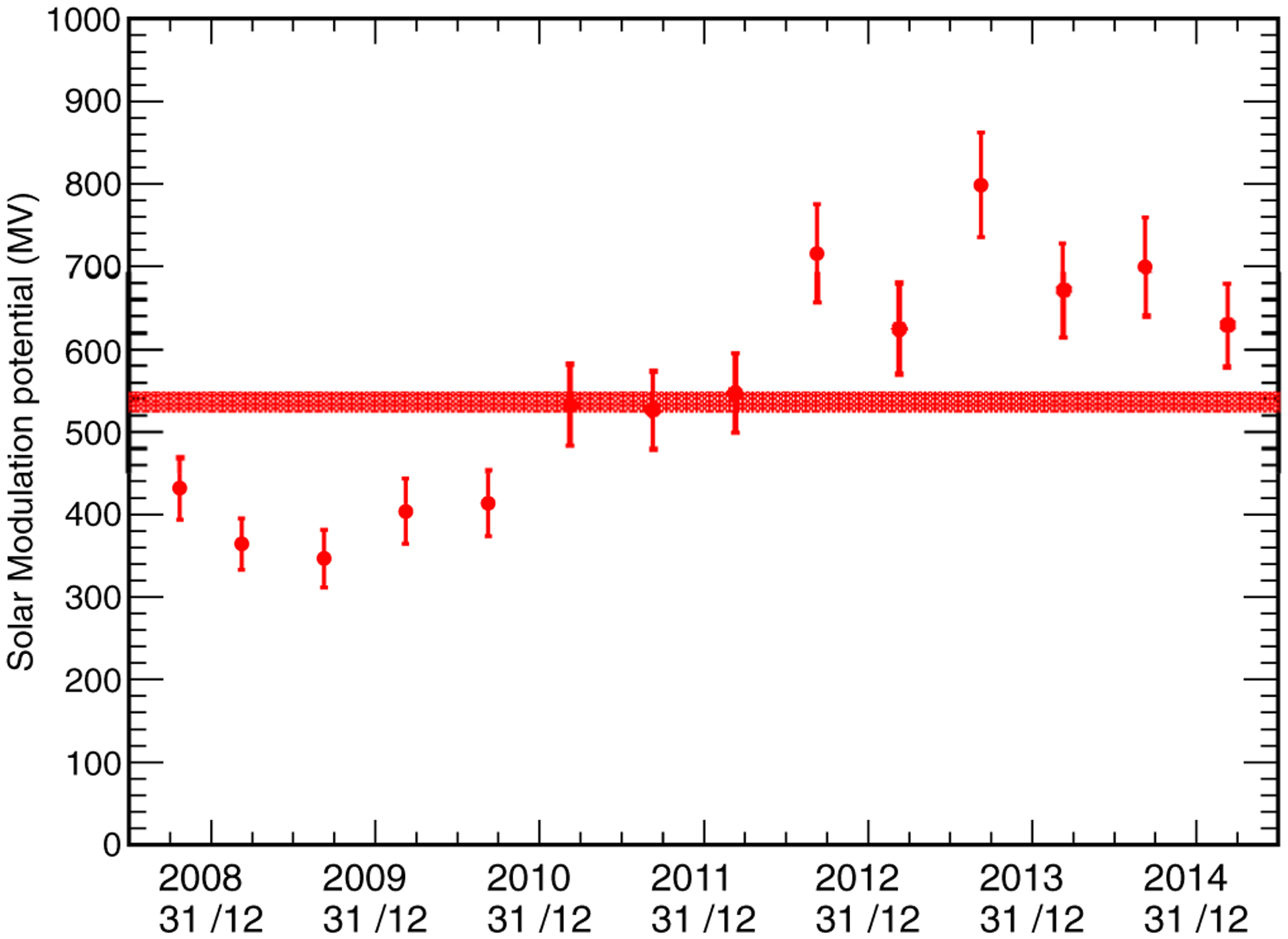
Time evolution of the solar modulation potential, evaluated from a fit of the lunar gamma-ray emission. The central band corresponds to the average value of the solar modulation potential during the whole data-taking period.

The fitting procedure discussed here was also applied to the 6 month data samples into which the original data set was divided, to study the time evolution of the solar modulation potential. Figure 11 shows the time evolution of Φ obtained from the fit. A comparison with the plots in Fig. 4 shows that, as expected, the value of the solar modulation potential is anticorrelated with the count rates of the various neutron monitors. It is also worth noting that, starting in the second half of 2012, the solar modulation potential oscillates about the mean trend from interval to interval. This feature might be due to the major solar flare activity in recent years.

## VII. CONCLUSIONS

We measured the fluxes of gamma rays produced by the interactions of charged CRs impinging on the surface of the Moon using data collected by the Fermi LAT from August 2008 to June 2015. Thanks to the high statistics of the data sample and to the newest version of the Fermi LAT event-level analysis and instrument response function, we have been able to measure the gamma-ray fluxes in an energy range that extends from 30 MeV up to a few GeV. The time evolution of the flux shows that the gamma-ray emissivity of the Moon is correlated with the solar activity.

We also developed a full Monte Carlo simulation of the interactions of CR protons and helium nuclei with the Moon using the FLUKA simulation code to evaluate the gamma-ray yields. We implemented two different composition models of the lunar surface and we found that the gamma-ray emission from the Moon depends on the elemental composition of its surface. In particular, we observe that the MP composition model provides a good agreement between the lunar gamma-ray data and the results of direct measurements of the CR proton and helium spectra.

Starting from a custom model of the CR proton and helium LIS, we then used the simulation to infer the local CR proton intensity spectrum from the Moon gamma-ray spectrum in the framework of the force field approximation. The CR spectra obtained with this procedure are consistent with the results from direct measurements performed by the PAMELA and AMS experiments. We applied this approach to evaluate the time evolution of the solar modulation potential. The results show that the potential is anticorrelated with the counts in several neutron monitors.

## Figures and Tables

**TABLE I.** Summary of the main features of the lunar surface composition models implemented in the simulation. The first panel shows the weight fractions of the different oxides composing the lunar surface. The second panel shows the value of mass density and the average values of the atomic number and of the mass number. The last panel shows the values of the radiation length and of the proton elastic and inelastic scattering lengths.

Model	Moskalenko & Porter, 2007	Turkevich, 1973
SiO_2_	45.0%	45.0%
FeO	22.0%	7.6%
CaO	11.0%	15.5%
A1_2_O_3_	10.0%	22.2%
MgO	9.0%	8.0%
TiO_2_	3.0%	1.1%
Na_2_O		0.6%
*ρ*(g/cm^3^)	1.80	3.01
〈*Z*〉	11.5	10.8
〈*A*〉	23.4	21.8
*X*_0_(g/cm^2^)	22.4	24.4
*λ*_*el*_(g/cm^2^)	84.5	82.1
*λ*_inel_(g/cm^2^)	150.4	148.4

## Footnotes

1 In the analysis of the background region, the Moon position is replaced with the position of the center of the background region.

2 Here, we define “bright” a source whose gamma-ray flux above 100 MeV is larger than 2 × 10^−7^photons cm^−2^ s^−1^.

3 The AMS-02 helium spectrum includes both ^4^He and ^3^He nuclei. Once again, it should be emphasized that we are considering the He primaries as consisting entirely of ^4^He.
